# Drying of Pigment-Cellulose Nanofibril Substrates

**DOI:** 10.3390/ma7106893

**Published:** 2014-10-01

**Authors:** Oleg Timofeev, Katariina Torvinen, Jenni Sievänen, Timo Kaljunen, Jarmo Kouko, Jukka A. Ketoja

**Affiliations:** VTT Technical Research Centre of Finland, P. O. Box 1000, FI-02044 VTT, Finland; E-Mails: oleg.timofeev@vtt.fi (O.T.); katariina.torvinen@vtt.fi (K.T.); jenni.sievanen@vtt.fi (J.S.); timo.kaljunen@vtt.fi (T.K.); jarmo.kouko@vtt.fi (J.K.)

**Keywords:** cellulose nanofibril, substrate, drying, evaporation, surface, material property

## Abstract

A new substrate containing cellulose nanofibrils and inorganic pigment particles has been developed for printed electronics applications. The studied composite structure contains 80% fillers and is mechanically stable and flexible. Before drying, the solids content can be as low as 20% due to the high water binding capacity of the cellulose nanofibrils. We have studied several drying methods and their effects on the substrate properties. The aim is to achieve a tight, smooth surface keeping the drying efficiency simultaneously at a high level. The methods studied include: (1) drying on a hot metal surface; (2) air impingement drying; and (3) hot pressing. Somewhat surprisingly, drying rates measured for the pigment-cellulose nanofibril substrates were quite similar to those for the reference board sheets. Very high dewatering rates were observed for the hot pressing at high moisture contents. The drying method had significant effects on the final substrate properties, especially on short-range surface smoothness. The best smoothness was obtained with a combination of impingement and contact drying. The mechanical properties of the sheets were also affected by the drying method and associated temperature.

## 1. Introduction

The growth of the printed electronics market is based on new low-cost materials and products. Recently, novel bio-degradable cellulose nanocomposite substrate materials have been introduced that compete well in price and material properties against oil-based plastic substrates [1,2]. Using cellulose nanofibrils (CNF) as the strength additive in the inorganic composite structure, it is possible to surpass the properties of traditional paper substrates. It has been shown that this new pigment-cellulose nanofibril (PCN) substrate can contain up to 90% of inorganic fillers and, yet, remain mechanically stable and flexible [1,3].

The composite structure includes a large proportion (approximately 20%) of CNF, which makes dewatering after structure forming very difficult, as CNF has a high water retention capacity [4]. During the development of the substrate, the drying was done in an oven at 70–80 °C. The drying process was very slow. In this work, we look for an optimal way of removing the water. Here, one should take into account both the drying efficiency and the effect of the drying method on the final product properties.

The drying of cellulose nanofibrils has been studied earlier using different methods, such as air drying, freeze drying, spray drying and supercritical drying [5,6]. Usually, the aim has been to preserve the morphology of the cellulose nanofibrils. Air drying of CNF suspensions forms a tightly packed material [6], and this drying method is often disregarded in applications where the nanostructure is essential. However, for the PCN substrate, tight packing of nanofibrils is beneficial in forming a smooth surface. On the other hand, a tight surface layer slows down the evaporation of water from inside the bulk material and may reduce drying efficiency. Thus, it is essential to determine this efficiency and material properties for drying methods that are close to air drying.

We study several available drying methods and their combinations that are in use in paper and board technology. In these industries, water from the web after the forming section is removed in the press section and the subsequent drying section. The amount of water removed by pressing is a function of applied pressure, nip width (dwell time) and water viscosity [7]. Mechanical dewatering is a much cheaper way to remove water than drying based on evaporation. After wet pressing, the typical solids content of wet web is 45%–55%.

The commonest drying methods for paper, board and cellulose are cylinder (contact) drying and air float and air impingement drying. Usually, a low steam pressure is used in cylinder dryers, and the drying rates are not very high: 10–25 kg/m^2^/h [8,9]. Air impingement dryers can operate at high air temperature (300–500 °C) and air velocity (50–100 m/s). An evaporation rate of 100 kg/m^2^/h is a common level for this type of dryer [10]. Another very effective method for removing moisture and improving mechanical properties is press drying. Press drying combines elements of pressing and hot surface drying. Condebelt drying represents one type of press drying, *i.e.*, drying under pressure and totally restrained [11]. Drying rates of 100–200 kg/m^2^/h are possible, depending on the process parameters [12].

This investigation was concentrated on determining the final PCN substrate properties and the drying rates for contact drying, impingement drying and press drying at moderate temperatures. Despite the large amount of bound water, these conventional drying methods turned out to be quite effective also for the PCN structure. Properties of the PCN sheets after drying and calendering were measured. We found a relatively large variation in the measured surface and mechanical properties depending on the drying method.

## 2. Materials and Methods

### 2.1. Materials

PCN substrate was prepared with a mixture of kaolin as a filler and CNF as a binder. The CNF samples were made from Finnish once-dried bleached hardwood (birch, *Betula* L.) The fibril cellulose prepared at VTT was obtained after eight passes through a Masuko Sangyo (Supermasscolloider type MKZA10-15J, Masuko Sangyo Ltd., Kawaguchi, Saitama, Japan) grinder by using a decreasing gap width and increasing operating power. The rotation speed was set at 1500 rpm. The CNF amount was 20 mass% and the initial consistency (percentage of solid, filterable material in suspension) was 3.5%.

The kaolin pigment used was Intramax 60 from Imerys. The kaolin amount was 80 mass%.

PCN sheets were cut from the webs manufactured on a VTT pilot machine, SutCo (Coatema Coating Machinery GmbH, Dormagen, Germany). The web was formed by the application of a 7% wet PCN mixture on a thin plastic PET film by the solvent casting method. The film thickness was 23 µm. In order to improve the release properties, the film was given the N-Ar plasma treatment before forming the structure. After the A4-size sheets were cut, they were lightly pressed using a metal plate and filter paper and sealed in carrier plastic bags at room temperature. The grammage of the final wet PCN sheets varied from 150 to 220 g/m^2^, and there was a similar variation in the initial solids content in the range of 20%–35%. This level of solids content after vacuum filtering has been found earlier in dewatering studies of similar PCN furnishes [4].

Reference board samples were cut from the never dried web made on a Metso pilot machine with a grammage of 106 g/m^2^ and initial solids content close to 50%.

### 2.2. Experimental Procedure and Characterization

In all drying and pressing experiments, the thin PET film used in the pilot production was kept under the wet sheets, so that drying or dewatering was always one-sided, *i.e.*, through the top side of the sheet. In order to prevent thermal degradation of the nanofibrils [5], all drying tests were performed at moderate temperatures.

The moisture ratio *MR*_i_ of the sample was determined by using the initial mass, measured before each drying step *m*_i_, and the final one *m*_d_, measured after keeping the sample in the oven for 2 h at an air temperature of 105 °C after the drying test was complete:

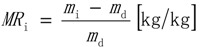
(1)

After drying, the PCN sheets were conditioned under a standard climate (25 °C, 50% relative humidity) before calendering and physical testing. Hot nip calendering is a standard operation in paper making by which the surface smoothness can be increased further. The calendering was done with approximately 20 MPa pressure and 150 °C temperature.

Standard characterization methods were used, except for the surface roughness measurement and mechanical testing of the samples. The grammage of the samples was determined according to [13]. The thickness was determined according to [14], and the density was determined based on the measured values of grammage and thickness.

Surface roughness was measured using an Altisurf 500 profilometer (Cotec, Évian, France) with a sampling interval of 1 µm × 1 µm and a total measured area of 1 mm × 1 mm. The results were filtered with a Gaussian 0.25-mm sized screen. The surface roughness was defined as:

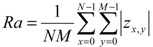
(2)
where *z_x_*_,*y*_ was the distance of the surface point to the mean surface height. The measurement was repeated nine times for each sample.

The mechanical properties of the PCN sheets were measured with the so-called C-Impact tensile tester with a sample length of 50 mm and a width of 15 mm [15]. The elongation speed was 10 mm/s (the strain rate was 20%/s). The frequency of data recording was 2 kHz. From the measured stress-strain curve, the strength and breaking strain were determined as the values corresponding to the maximal stress. The elastic modulus was obtained as the maximal slope of the tangent to the stress-strain curve. The test was repeated five times for each trial point.

### 2.3. Drying Methods

Drying was investigated for three methods and their combinations that are in use in paper and board technology: contact drying, impingement drying and press drying. All of these methods are able to form a compact surface for the PCN structure.

**Figure 1:**
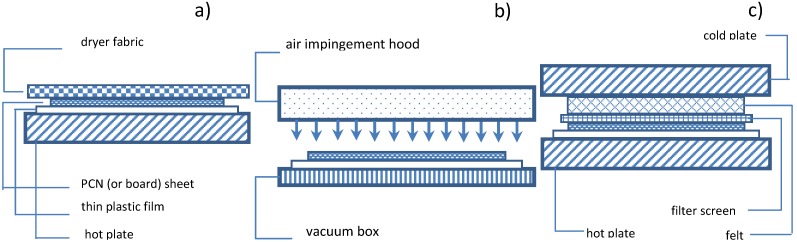
The main components of the studied drying methods: (**a**) contact dryer; (**b**) air impingement dryer; (**c**) hydraulic press. PCN: pigment-cellulose nanofiber.

The contact dryer is shown schematically in Figure 1a. It includes a curved metal plate, heated by electrical coins from the bottom side, and a tensioned dryer fabric. Experiments were carried out at a hot plate temperature of 50 and 80 °C and a constant fabric tension of 2 kg/cm. A commercial dryer fabric made from flat yarns and with a permeability of 1600 m^3^/m^2^/h was used. Each paper sample with thin plastic film was weighed, positioned on top of the hot plate and covered with the fabric. The timing of the drying trial began at this point and continued until the fabric was lifted up and the sheet removed from the hot plate for the second weighing.

The air impingement dryer is shown in Figure 1b. The dryer consists of an air impingement hood, a vacuum box and the air heating system. The air impingement hood has impingement geometry matching commercial dryers, with similar open area, nozzle distances, nozzle diameter and distance to the wet material. Every wet sheet on the thin plastic film was placed on the vacuum box and moved under the impingement hood. Under the hood, the vacuum box with the sample was moving back and forth with a special driving mechanism to provide uniform drying conditions.

In hot pressing experiments, a hydraulic laboratory sheet press was used with an additional hot bottom plate (Figure 1c). Tests were carried out with two pressures: 60 and 420 kPa. A steel plate with a size of 160 × 160 mm^2^ and a thickness of 25 mm was used as a bottom press plate, which was pre-heated before each test. The temperature of the plate was 80 or 110 °C.

## 3. Drying Kinetics

**Figure 2:**
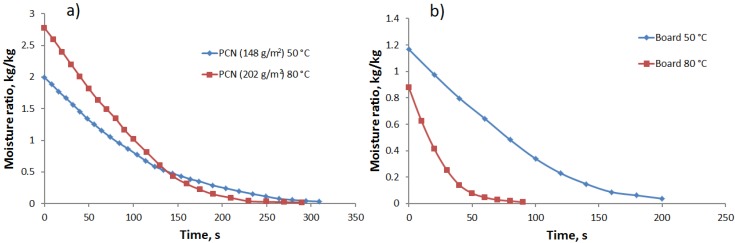
Drying kinetics for the contact drying of (**a**) PCN and (**b**) board sheets at two different temperatures: 50 °C (blue) and 80 °C (red).

The initial moisture ratio of the PCN sheets before drying is approximately 2–3-times higher than that of the board samples, in the range of 2.5–3.8 kg/kg. Assuming all of the water to be bound to the CNF with 20 mass%, this corresponds to 13–19 kg water/kg CNF. This level agrees well with the water retention values measured earlier for wet CNF [4]. Moreover, the grammage of the PCN sheets is also almost 1.5–2-times higher. Both factors increase the drying time required for complete drying of the PCN sheets in comparison with board samples. This is seen in the drying curves of the contact drying tests shown in Figure 2 for the PCN substrate and board samples. For example, at a hot surface temperature of 80 °C, the total drying time of the PCN sheets is about 220 s, and for the board samples, it is less than 90 s.

**Figure 3:**
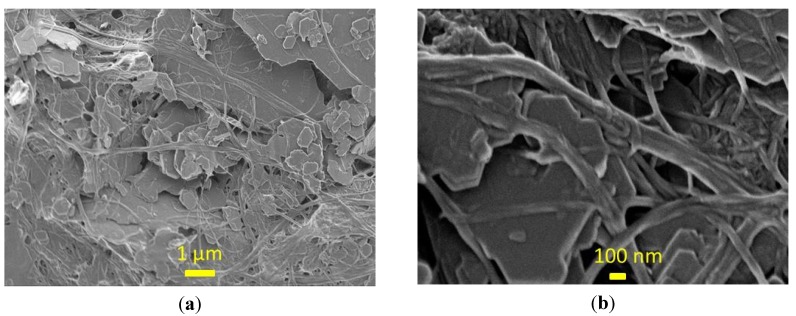
High resolution SEM images of the PCN sheet before calendering. The cellulose micro-“membranes” seen at a lower resolution between the kaolin pigment particles (**a**) are actually porous fibrillar networks in the nanoscale (**b**).

All drying curves in Figure 2 indicate two phases: the constant rate phase and the falling rate phase. In the constant rate phase, moisture removal is dominated by the surface evaporation rate from the wet sheets containing significant amounts of free water [16,17]. In the falling rate phase, the drying rate decays with time, as residual moisture requires more time to be removed from the interior of the sheet. The moisture content at which the constant rate phase ends is for the PCN sheets close to 1.0 kg/kg, corresponding to 5.0 kg water/kg CNF, if we assume all water to be bound to CNF. This can be compared to the transition to the falling rate phase in the range of 0.5–0.6 kg/kg for the board sheets, which is a typical maximal bound water content in mechanical wood fibers [16,17]. In other words, the bound water content in CNF can be an order of magnitude larger than in the board when the transition between different drying phases takes place. This huge difference would mean that internal moisture transport would limit the drying rate at much higher cellulose water content for the PCN substrates than it does for the board. In fact, the cellulose structures of the two types of samples are quite different. In the PCN sheets, CNF gel agglomerates during drying to micro-membranes/networks that bind filler particles together [1], as shown in Figure 3. The forming agglomerates within inter-particle pores can begin to limit the moisture transport at a relatively early drying stage. On the other hand, even dry membranes contain nano-scale pores that allow effective vapor diffusion. Therefore, the total drying time is reasonable compared to the board where the slow diffusion [18,19] in the fiber walls is the main cause for the decaying drying rate at low moisture ratios.

The above reasoning can be justified by comparing the diffusion constants in various parts of the structure. The vapor diffusion constant in the pore space of normal board is of the order of 5 × 10^−7^ m^2^/s [19]. This can be compared with the moisture diffusion constant in the fiber wall. This constant has a maximum level of 2 × 10^−10^ m^2^/s for fully water-saturated fiber wall, and the diffusion constant decays exponentially with the increasing solids content as inter-fiber pores close up [18,19]. The exponentially decaying diffusion rate explains the strong falling-rate phase observed in Figure 2b for the board. On the other hand, the decay is not as strong for the PCN structure, because of the significant vapor diffusion in the remaining nano-scale pores of the CNF networks. Experimental studies [20] have shown that this diffusion is not significantly slower than the one observed in normal wood fiber networks.

**Figure 4:**
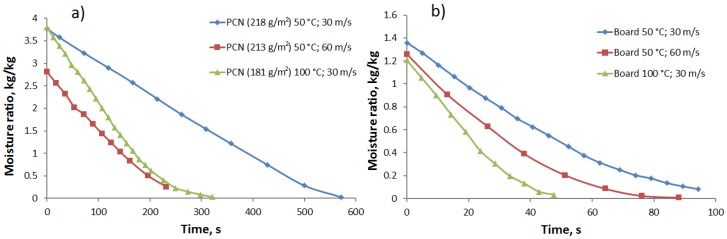
Drying curves for the air impingement drying for (**a**) PCN sheets and (**b**) the board at three different air speed and temperature conditions.

For air impingement drying, the development of the moisture ratio is shown in Figure 4 for three combinations of air jet velocity and air temperature for both types of samples. The transition from the constant drying rate phase to the falling rate phase takes place at similar moisture ratio levels (1.0 kg/kg for the PCN sheets and 0.5–0.6 kg/kg for the board), as in the case with contact drying. On the other hand, the difference in the total drying time between the PCN sheets and the board is slightly larger in this case than for the contact drying. This is partly explained by the high initial moisture content of the studied PCN sheets; see Figure 4a.

**Figure 5:**
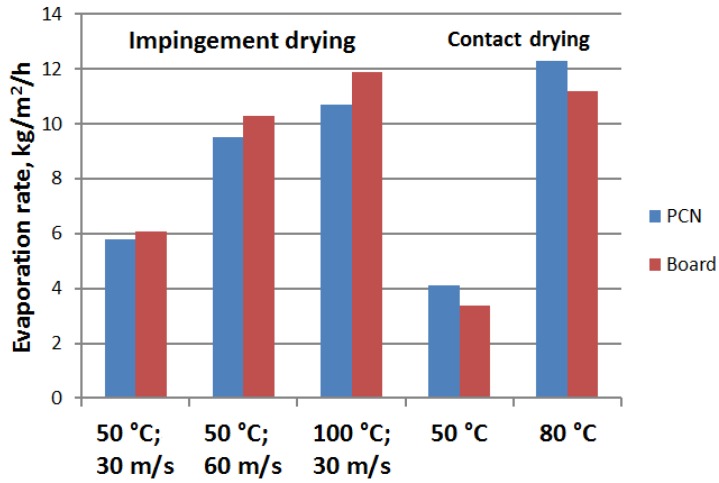
Drying rates determined from the initial linear part of the drying curve for the air impingement and contact drying methods for both PCN sheets and board in varied drying conditions (temperature and air speed).

Figure 5 shows that there is actually no major difference in the initial drying rates for the two types of samples. The lowest and highest drying rates are observed for the contact drying method for both types of samples. The air impingement method leads to intermediate values in between the above two extremes. In general, drying rates measured for the PCN sheets and board samples are quite comparable at the same drying parameters. In the first drying phase, the drying rate is controlled by external drying conditions, and this rate depends less on material properties. The smooth surface of the PCN substrate helps in contact drying as compared to the board. On the other hand, air impingement appears to be more effective in removing free water from the pores of the board than from the denser PCN structure. The latter distinction is enhanced in the falling drying rate phases of Figure 4, where air speed affects the drying more for the PCN structure than what is observed for the board. Due to the high permeability of the board, air is able to penetrate into the board more easily than through the CNF gel of the PCN sheets.

**Figure 6:**
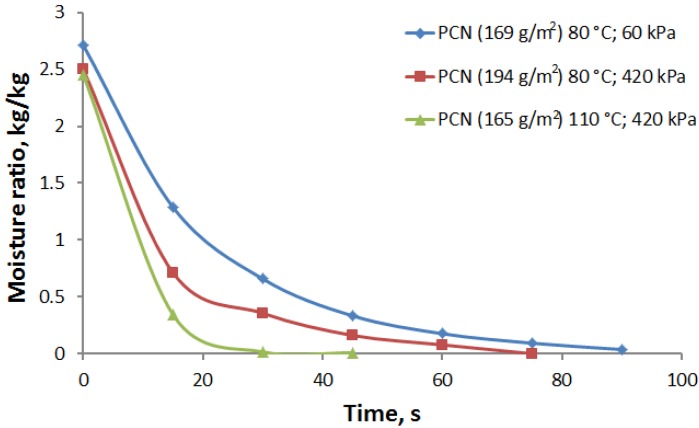
Moisture ratio *vs.* time for PCN sheets in hot press dewatering for varied pressure and temperature.

The hot press dewatering curves are shown in Figure 6. The effect of pressure and temperature on dewatering time is clearly seen. Dewatering curves can be divided into two phases. In the first phase, the dewatering rate is very high until a moisture ratio of 0.75–1.0 kg/kg is reached. In this phase, water is mainly squeezed out from the sheet under the applied pressure. In the second phase, the dewatering rate is much lower due to slower evaporation and diffusion processes.

At a hot plate temperature of 80 °C, an increase in pressure from 60 to 420 kPa reduces the first-phase dewatering time to almost one half, *i.e.*, from 30 to 15 s when coming down from the initial moisture ratio to 0.75 kg/kg. Moreover, the dewatering rate can be increased further using a higher temperature due to the lower viscosity and surface tension of the water [7,21]. For example, if the temperature is increased from 20 to 90 °C, viscosity is reduced by a factor of three from 1.0 to 0.3 cP. The surface tension is also reduced, but not as much as the viscosity. The effect of these reductions is seen in Figure 6 at a plate temperature of 110 °C. If the moisture content of a PCN sheet is reduced from 2.5 kg/kg down to 1.0 kg/kg by hot pressing, the amount of water that needs to be evaporated during the second phase is reduced by 60%. This results in a dramatic reduction in energy consumption for drying.

**Figure 7:**
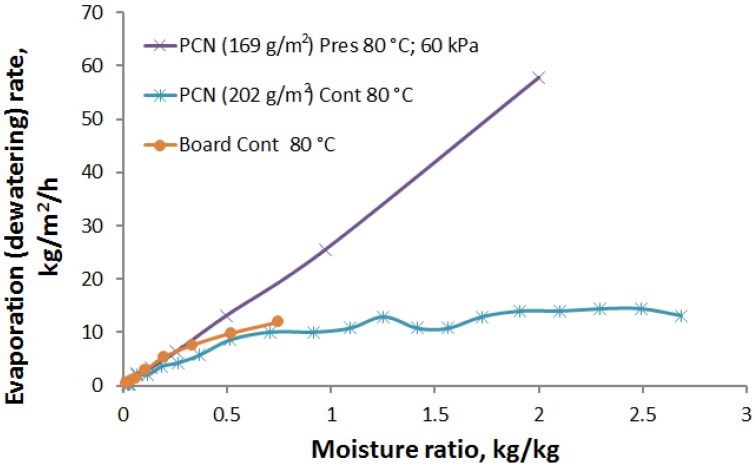
Comparison of the press dewatering rate (pressure 60 kPa, temperature 80 °C) with the evaporation rate in the normal contact drying (equal temperature) for the PCN sheets. The corresponding result for the contact drying of board is shown by the dots.

In the second phase of the drying process, for moisture contents below 0.75–1.0 kg/kg, the drying can be significantly accelerated by a temperature increase from 80 to 110 °C. In this region, mere mechanical pressing does not help to remove water any longer. Residual moisture is removed more effectively by evaporation. Increasing the temperature of the moist sheet results in an exponential increase of the vapor pressure. In Figure 6, the temperature increase from 80 to 110 °C (at constant pressure 420 kPa) reduced the second-phase drying time by more than a factor of two. Figure 7 shows a comparison of the press dewatering rate and evaporation rates for the contact drying method. At hot pressing, the dewatering rate has an initial maximum value of 60 kg/m^2^/h, which comes down almost linearly with decreasing moisture content. The evaporation rate in the contact drying is approximately 12 kg/m^2^/h for moisture ratios higher than 0.75 kg/kg. Below a moisture ratio of 0.5 kg/kg, the evaporation rates for both drying methods develop in almost the same manner and also follow the drying rates measured for the board. Thus, the main advantage of the press drying is to remove a major part of the moisture as a liquid without evaporation and to correspondingly reduce the drying time and energy consumption for drying. Moreover, mechanical pressing improves the contact heat transfer between the hot plate and the PCN substrate, which leads to faster evaporation in the beginning of the second-phase drying, as compared to contact drying (see Figure 7).

## 4. Effect of Drying on PCN Sheet Properties

The PCN substrate should meet certain quality requirements in order to be applied as a base for printed electronics. The main requirements concern the surface smoothness. The roughness level 0.3–0.4 μm (see Equation (2)) is sufficient for inkjet-printed conductors, screen-printed near-field communication RFID (radio-frequency identification) antennas and spin-coated thin transistors [22]. In addition, sufficient strength, flexibility and breaking strain of the substrate are required. We found significant effects from the drying process on these factors. The measured properties after calendering of the PCN sheets are shown in Table 1.

**Figure 8:**
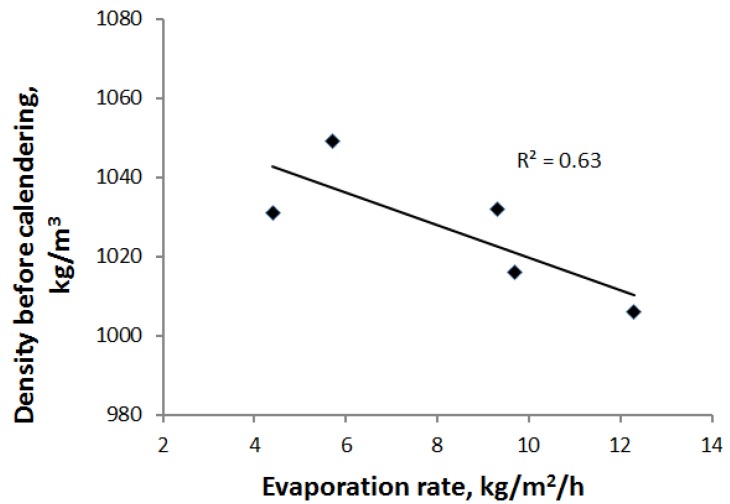
Sheet density before calendering is slightly affected by evaporation rate for contact and impingement drying.

For contact and impingement drying, the sheet density before calendering decreased slightly with the drying rate, as shown in Figure 8. The longer drying time seems to lead to the greater shrinkage of the PCN sheet in the thickness direction.

The pressure in the hard nip calendering process is typically 40–80 MPa, *i.e.*, significantly higher than the pressure we used in the laboratory hot press. The calendering operation did not reduce the slight density variations of the samples observed before calendering. Moreover, there was no correlation between the densities before and after calendering for the varied drying methods. For example, press drying gave the highest density before calendering and the lowest density after calendering. This suggests that different drying methods led to deviations in the microscopic composite structure and, therefore, to different overall deformations during calendering.

**Table 1:** Measured properties of the PCN substrates after calendering.

Drying method	Impingement			Contact		Impingement and contact	Press			
Temperature (°C)	50	50	100	50	80	50 (Impingement), 80 (Contact)	80	80	110	110
Air speed (m/s)	30	60	30	–		30	–			
Pressure (kPa)	–						60	420	60	420
Thickness (μm)	178	148	144	127	145	116	148	168	101	154
Density (kg/m^3^)	1,421	1,534	1,438	1,523	1,448	1,518	1,467	1,309	1,437	1,350
Roughness, top side (mm)	0.48	0.51	−	0.71	0.41	0.45	0.63	0.86	0.82	0.60
Roughness, bottom side (mm)	0.45	0.38	−	0.42	0.49	0.34	0.43	0.43	0.43	0.42
Tensile strength index (Nm/g)	13.4	16.6	16.0	12.5	15.8	15.8	12.4	14.3	17.5	15.6
Elastic modulus (GPa)	4.6	5.2	4.3	4.3	4.7	4.6	4.2	4.1	3.8	3.6
Breaking strain (%)	1.3	1.9	1.3	0.9	1.3	1.6	1.0	1.0	1.6	1.5

Quite surprisingly, press drying with a higher pressure level of 420 kPa led to a lower density after calendering than the lower pressure 60 kPa; see Table 1. The high pressure during press dewatering may prevent horizontal particle movement later during calendering, thus leading to reduced final density. On the other hand, the mechanical properties were dominated by pressing temperature rather than by final density. Higher tensile strength and breaking strain were observed with the higher pressing temperature of 110 °C, even though the elastic modulus was somewhat smaller in this case than at the lower pressing temperature of 80 °C.

**Figure 9:**
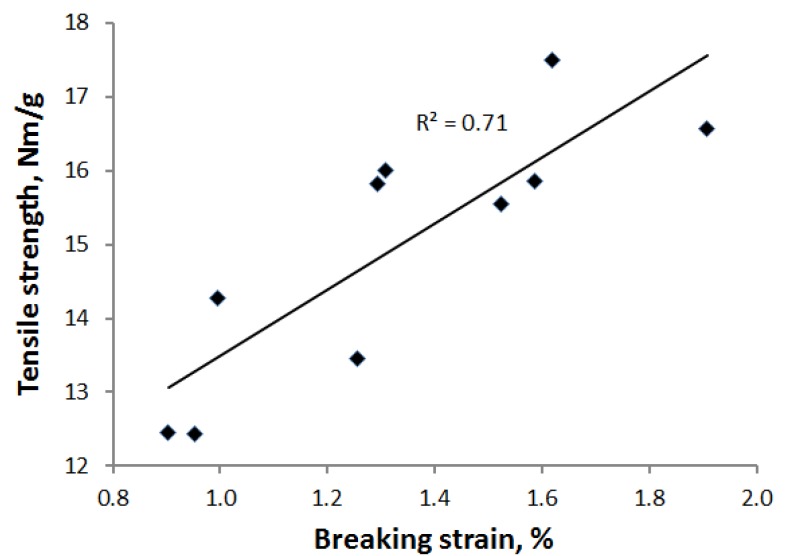
Correlation between strength and breaking strain for the calendered sheets obtained with the various drying methods.

**Figure 10:**
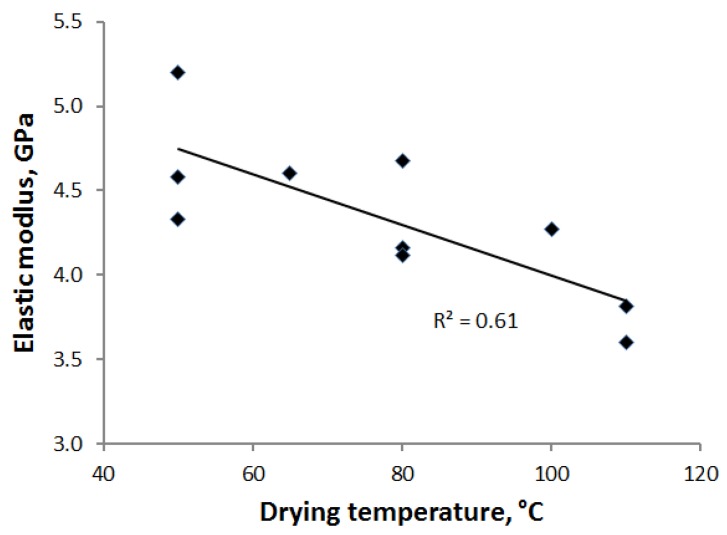
Elastic modulus of the calendered PCN sheets *vs.* drying temperature for all drying methods. For the combination of impingement and contact drying, the average temperature 65 °C (see Table 1) was used when plotting the figure.

There was a clear correlation between strength and breaking strain for all trial points, as shown in Figure 9. However, no such correlation was found between tensile strength and elastic modulus. The elastic modulus appeared to be affected by drying temperature, as indicated in Figure 10. At a high temperature, non-crystalline parts of the cellulose nanofibrils soften, and simultaneously, the drying stresses increase. These two factors may lead to significant structural deformations and permanent changes in mechanical behavior [17,23]. The way drying shrinkage is prevented may have an additional effect on the elastic modulus. Drying was most restricted for the press drying that also led to the smallest moduli. However, as mentioned earlier, temperature seemed to play a role for this method, as well.

The smoothness of the bottom surface against the plastic sheet was generally higher than that of the top surface. The only exception to this rule was found for the higher temperature (80 °C) contact drying, for which the top “open” surface was slightly smoother than the bottom surface after calendering. Before calendering, the order was normal also for this trial point. The difference in the roughness of the two surfaces was particularly marked for all press drying points, further suggesting that this drying method “freezes” the structure, causing it to be less moldable in calendering. Moreover, even for the smoother bottom surface for the press drying, the roughness was greater than the best results for the other drying methods.

The lowest roughness value of 0.34 μm for the bottom surface was observed for the combination of the contact and impingement drying. In this case, impingement drying was first carried out until the moisture ratio became roughly 1.0 kg/kg, after which contact drying was applied to obtain the final solids content. However, the drying parameters were not optimized, so that even better smoothness could be possible. Earlier, in [2], the surface roughness of the substrates formed with a different laboratory-scale process was measured after oven drying and calendering. The surface roughness was measured for both “fine” and “coarse” cellulose nanofibril grades using the same pigment type as here. The values obtained after calendering were 0.51 μm (fine quality) and 0.60 μm (coarse quality). The lowest roughness values obtained here are thus clearly better than the above values.

In [2], the small-scale roughness and porosity were also simulated on a particle level by looking at the packed structure formed by the skeleton of pigment particles. Such simulations led to a roughness value of 0.67 μm for the profilometer spot size of 1 μm used in the roughness measurements (the same as in the present study). The predicted value of 0.67 μm for pure kaolin packing is reasonably close to the overall experimental range, 0.34–0.60 μm, for the kaolin-cellulose nanofibril composite. However, this comparison makes it clear that cellulose nanofibrils make the surface smoother in the scale of the profilometer spot size. This scale is similar to the length of the nanofibrils. For slightly larger scales, the variation in roughness is quite mild, even for pure pigment surfaces, as shown in Figure 11 of [2]. The short-range surface smoothening effect is strongly affected by the drying method, leading to the above relatively large variation of the roughness values. The drying rate probably affects the size and density of the nanofibril clusters responsible for smoothing out the pigment surfaces and their edges.

## 5. Conclusions

In laboratory-scale experiments, it was shown that the PCN substrate can be dried using conventional drying methods, such as contact drying and air impingement drying methods. Despite the very large bound water content of the CNF gel, the estimated drying rates for the PCN substrate are comparable with the drying rates of typical board with the same external drying conditions. The CNF agglomerates seem to affect the moisture transport in an early drying stage, but for the low moisture ratios, nano-scale pores in the CNF membranes/networks speed up vapor diffusion, as compared to the moisture diffusion in the fiber walls within the board.

It was also demonstrated that, before drying, the water can be effectively removed from the PCN sheets by hot pressing. Removal of water mechanically in the hot press will dramatically reduce the energy consumption required for drying of an initially very wet PCN substrate.

The final properties of the PCN sheets after calendering could be significantly affected by the drying method. Unfortunately, hot pressing led to somewhat unconformable structures with poorer surface smoothness than with the other drying methods. The best smoothness of all of the drying methods was obtained with a combination of impingement and contact drying. Mere contact drying led to an almost equally smooth surface. The required smoothness level [22] for various applications, like inkjet-printed conductors, screen-printed near-field communication RFID antennas and spin-coated thin transistors, was achieved with both methods. By fine tuning the forming and drying processes (e.g., temperature, air speed), the substrate properties could be probably further optimized. In this development, one should take into account that the drying method and temperature affect the mechanical properties of the PCN sheets, in addition to the surface properties.

## References

[1] Torvinen K., Sievänen J., Hjelt T., Hellén E. (2012). Smooth and flexible filler-nanocellulose composite structure for printed electronics applications. Cellulose.

[2] Penttilä A., Sievänen J., Torvinen K., Ojanperä K., Ketoja J.A. (2013). Filler-nanocellulose substrate for printed electronics: Experiments and model approach to structure and conductivity. Cellulose.

[3] Rantanen J., Lahtinen P., Maloney T. (2013). Property space for fibre, microfibrillar cellulose and precipitated CaCO_3_ composite sheets. Int. Pap. IPW.

[4] Dimic-Misic K., Puisto A., Paltakari J., Alava M., Maloney T. (2013). The influence of shear on the dewatering of high consistency nanofibrillated cellulose furnishes. Cellulose.

[5] Peng Y., Gardner D.J., Han Y. (2012). Drying cellulose nanofibrils: In search of a suitable method. Cellulose.

[6] Peng Y., Gardner D.J., Han Y., Kiziltas A., Cai Z., Tshabalala M.A. (2013). Influence of drying method on the material properties of nanocellulose I: Thermostability and crystallinity. Cellulose.

[7] Walker K. (1990). Advances in hot pressing technology. Tappi J..

[8] Timofeev O., Belski A., Mujumdar A., Mujumdar A.S. (1999). Effect of dryer fabric pressure on multi-cylinder drying of paper. Drying of Solids.

[9] Asensio C., Seyed-Yagoobi J., Lehtinen J., Karlsson M., Timofeev O., Juppi K. (1995). Comparison of several multi-cylinder paper drying simulation models. Dry. Technol..

[10] Kiiskinen H., Juppi K., Timofeev O., Karlsson M., Edelmann K. (1999). Impingement drying of multi-ply linerboard. Pulp Pap. Can..

[11] Lehtinen J. (1995). Condebelt drying of paper and paperboard for optimizing quality and production for many grades. Dry. Technol..

[12] Timofeev O., Ilomäki J., Kuusela J. Effect of process parameters on paper temperature in Condebelt drying. Proceedings of the 14th International Drying Symposium (IDS 2004).

[13] (1995). Paper and Board—Determination of Grammage.

[14] (1998). Determination of Thickness and Bulk Density or Apparent Sheet Density.

[15] Lipponen P., Kouko J., Leppänen T., Hämäläinen J. (2008). Elasto-plastic approach for paper cockling phenomenon: On the importance of moisture gradient. Int. J. Solids Struct..

[16] Weise U., Maloney T., Paulapuro H. (1996). Quantification of water in different states of interaction with wood pulp fibres. Cellulose.

[17] Niskanen K. (2008). Paper Physics.

[18] Topgaard D., Söderman O. (2001). Diffusion of water absorbed in cellulose fibers studied with 1H-NMR. Langmuir.

[19] Gupta H., Chatterjee S.G. (2003). Parallel diffusion of moisture in paper. Part 1: Steady-state conditions. Ind. Eng. Chem. Res..

[20] Huang B., Hill R., van de Ven T. (2012). Nanopaper: Thin films prepared from polymeric nanotubes. Macromol. Mater. Eng..

[21] Paulapuro H. (2008). Papermaking Part 1, Stock Preparation and Wet End.

[22] Torvinen K., Sievänen J., Hassinen T., Mattila T., Hellén E. Flexible pigment-nanocellulose substrate for printed electronics with good thermal tolerance. Proceedings of the 1st International Symposium on Nanoparticles/Nanomaterials and Applications.

[23] Paavilainen S., McWhirter J.L., Róg T., Järvinen J., Vattulainen I., Ketoja J.A. (2012). Mechanical properties of cellulose nanofibrils determined through atomistic molecular dynamics simulations. Nordic Pulp Pap. Res. J..

